# Setting the agenda in environmental crisis: Relationships between tweets, Google search trends, and newspaper coverage during the California drought

**DOI:** 10.1371/journal.pone.0259494

**Published:** 2021-12-07

**Authors:** Sorin Adam Matei, Robert Kulzick, Valeria Sinclair-Chapman, Lauren Potts

**Affiliations:** 1 Brian Lamb School of Communication, Purdue University, West Lafayette, IN, United States of America; 2 PSB Market Research, Washington, DC, United States of America; 3 Department of Political Science, Purdue University, West Lafayette, IN, United States of America; 4 Michigan State University, East Lansing, Michigan, United States of America; Universitat de Barcelona, SPAIN

## Abstract

Nuanced public responses to droughts and other chronic environmental crises reflect today’s increasingly complex communication ecosystem. At once global and infinitely customizable, this vast array of media and information channels requires existing theory to address the implications of interactions among social media, “traditional” mass media outlets, and information-seeking tools such as search engines. How do these channels intervene in public conversation? What might the agenda-setting perspective have to say? Data collected during peak years of the California drought, 2013–2015, indicate that California residents responded to worsening drought conditions Twitter first, which was the only media behavior directly stimulated by environmental stressors. Google searches stimulated newspaper coverage and Twitter activity, revealing the centrality of search behaviors in this environmental crisis. The findings suggest significant changes to the communication landscape as individual and collective users become increasingly dependent on non-mainstream media channels for information in chronic crisis situations.

## Introduction

Natural crisis situations generate an enormous amount of public interest across social demographics. Everyday citizens who are aware of or affected by the situation exhibit intensified information-seeking behaviors [1]. Commercial media channels implement crisis coverage with an eye toward increasing audience reach and engagement [2]. Policy makers deploy communication and mobilization campaigns in hopes of solving the crisis before the accompanying deluge of public discontent. Rapid segmentation of audiences according to communication objectives and crisis proximity entails rapid proliferation of communication agendas [3]. Given the speed of online information production and transmission—heightened even more by the chaos surrounding generation and circulation of crisis-related information—understanding where information starts and where it goes is more complex than ever. Our contribution considers the role continues chain of events, from a natural stressor (in this situation a drought), to social media early responses, to media amplification and further feedback. Our agenda setting model adds a continuous model of interaction stretching over many months that considers continuous feedback between social media, seen as a “first mover” and traditional media. The theoretical and empirical rationale for our model as well as its points of departure from existing research are presented below.

Our work starts from the premise that far from being an issue of purely academic interest, agenda-setting in times of crisis has practical implications for public conversation and governmental policy, as demonstrated by the current discussions about new grand strategies aiming to handle societal crisis, such as the “Green New Deal.” The public policy space was fundamentally shifted by the mere introduction of the term, switching public approval for the policy from the negative to the positive (more people agreeing that not with it) within a couple of years [4]. Timely and well-informed responsiveness to information flow is critical for stakeholders on all sides of a crisis. What is discussed determines what is done [3]. In consequence, charting the trajectories of discussion and decision-making processes becomes critical. To be sure, tracing how agendas are prioritized and enacted is not an easy task. Existing literature demonstrates that the media agenda interacts with and amplifies the emergent agenda spawned by offline and online conversations between everyday citizens [5–8]. Future research must attempt to penetrate the communication ecosystem connecting agendas set by one-to-many (i.e., broadcast, traditional, or print channels), many-to-many (i.e., social media, computer-mediated communication), and face-to-face (i.e., interpersonal) interactions. In times of crisis, especially, information travels from sub-network to sub-network through person-to-person, socially mediated, and traditional journalistic channels. In traversing these sub-networks, crisis messages may themselves be shaping crisis situations. This paper chronicles the order in which communication channels responded to a real-life, chronic crisis, the California drought of 2013–2015. We attempt to identify the communication factors that served as an early response system and helped set the tone of public conversation. While this is an agenda-setting study, we aim still higher, exploring the phenomenon in a naturalistic context with immediate social relevance. We speak directly to practical interactions between human and environmental contexts and processes, outlining the complete flow of information, starting with that about the natural environment and ending with the interaction between social and traditional media. These are distinguishing features for our project, which were not combined in this way in the previous work.

Research in a naturalistic crisis must also address the associated methodological challenges that necessitate innovative approaches to data collection and analysis [7]. Observing public reaction to societal challenges via social media requires the processing of massive amounts of information, which poses a significant methodological obstacle. Data collection needs to be comprehensive and rapid. Statistical tools capable of detecting the order of precedence in a noisy information environment must also be identified. The present study tackled these challenges by employing a data science approach [7]. We employed large-scale data harvesting from traditional media archives, social media streams, and archives of general web activity. Discrete human communicative behaviors, including more than 186,149 tweets, 1,853 articles, and an index of Google searches relevant to the drought, were pinpointed and classified by time, location, authorship, and usage pattern. The data provided solid footing from which to address the question of whether conversations about this chronic and deepening water crisis were more or less likely to start with traditional media (newspapers), social media (Twitter), or more generic information-seeking tools (search engines). In terms already consecrated in mass-communication research, we asked the linked questions: 1) Is there a complete chain of information in chronic cycles, from natural stressors to 1) Who or what responds first to an environmental crisis? 2) Who or what starts the news cycle, and 3) What role do social media users play vis-à-vis traditional media channels?

Our study operates within a theoretical framework documented by emerging research that suggests that a hybrid mass-personal media space has developed, with social media continuing to increase in influence [9]. The more recent literature also suggests specific intermediate interactions [5, 6], as well as momentary effects [8], which we take into account. However, existing research is far from definitive. Both the older [10] and more recent literature [5, 8] also indicates that mass media remains an important driving force in the processes of content creation and dissemination, wherein social media serves the subordinate role of message amplifier [11] for traditional media outlets. This paper seeks to contribute clarity to understandings of the order of precedence in longer cycles of natural stress and chronic crisis and the specific role of social media as a connector between environmental signals and public conversation.

By concentrating on communicative responses to drought, a chronic natural crisis comparable to pollution, climate change, and loss of habitat, we target a broader literature gap. Current crisis literature overwhelmingly concerns acute, immediate, event-based crises rather than chronic, long-term natural emergencies [12] Chronic crises may evolve quite slowly over time and often see their impact debated along political lines. These extended periods of time often engender decreased public interest in and sensitivity to the crisis and pose the challenge of massive data loss due to temporal attrition of information. Further, tracking media interactions across long periods of time constitutes a significant obstacle in and of itself: Data is heterogeneous in nature and the passage of time is inevitably accompanied by increased information noise.

In sum, the present study undertakes a re-examination of the agenda-setting hypothesis in the context of the recent (2013–2015) California drought, attempting to determine the way signals coming from the natural environment are interpreted by and reflected in communication channels at a variety of levels. We investigate whether, in the region of California most affected by the drought, public conversation was initiated by traditional (newspaper) media, social media (Twitter status updates), or the broader information-seeking strategy of using a search engine (Google). This focus on macro rather than micro agenda-setting processes illuminates the order in which communication elements respond to a crisis and respond to each other, leaving analysis of individual cognitions or evaluative choices to future research. In this respect, our study builds on but also uses a longer longitudinal perspective than previous studies [8]. Our macro focus includes examination of the role played by information-seeking tools, such as search engines.

Several previous studies looked at the role search engines played in disseminating misinformation [13], capturing public debate issue salience [14] or at the interaction between searches and the coverage of natural disasters [15]. Public debates occasioned by political scandals were also investigated [16], but with a focus on how search engines capture the public mood, rather than as direct predictors. Overall, prior research focuses mostly on the relationship between media and search trends seen as a pair [13, 16]. The triangular relationship between traditional or social media issue coverage is far less represented in the literature, especially in the case of chronic natural stressors. The agenda-setting research space still needs more evidence about the role of search engines in shaping public conversations about valid and urgent matters as it connects traditional and social media. Rojecki and Meraz [13] opened the way to looking at the temporal role search engines play in the chain of effects that lead to media conversations. We not only continue, but generalize this work, but looking at the spread of general information (not only misinformation) and by connecting social, traditional, and search media in a more integrated ecosystem of interactions.

Our longitudinal approach tracks the interplay of communication elements for 132 weeks (33 months, or 2 years and nine months, February 2013-September 2015) of the water crisis. For the Central Valley area of California, more specifically San Joaquin Valley, we analyze not only media consumption, production, and information-seeking data, but also the physical and hydrological data that ground our investigation.

In the following sections, we describe the natural, social, and political contexts of the water crisis and articulate a macrosocial theoretical framework for understanding the agenda-setting processes at play. Our research questions, writ large, focus on the way mass-media consumption, social-media participation, and online information-seeking intersect with a major environmental crisis. The “big dataset” facilitates investigation into the order of precedence of the communication elements in this crisis conversation. Findings speak not only to technical phenomena but to the possibility that, in times of chronic crisis, new information and communication patterns might foster new means of detecting human responses to environmental stress signals.

### The California drought

The California Drought provides challenging and yet fertile ground for macro-level agenda-setting research in the context of environmental crisis [17]. As the drought escalated over several years, communication channels had sufficient time to develop discrete flows of information that are traceable individually and as parts of an information ecosystem. The drought was long in coming—California saw chronic water shortages for more than three decades [18]. However, the disappointing 2012–2013 rain season bore the most tangible signs of drought to date. By 2013, the water supply in California had begun a dramatic decline due to chronic shortages. Shortages worsened in 2014 as California received a mere 49 percent of the historical average precipitation. State reservoirs ended water year 2014 at just 57 percent of their historical average, rendering it impossible for the existing water supply to sustain all existing needs. California Governor Jerry Brown declared a State of Emergency on Jan. 17, 2014, citing low levels of rainfall from 2012 onward and projected low levels of precipitation for 2014. Small wonder, then, that the water year ending in September 2014 proved to be the third driest on record [19].

The drought continued into 2015. According to a preliminary study by the University of California, Davis, the drought cost California $2.7 billion and 18,600 jobs in 2015 in addition to the estimated $2.2 billion loss in 2014 [20]. Long-term costs of the drought include an increasing depletion of groundwater that handicaps California’s ability to manage subsequent droughts. While agriculture was most dramatically affected, other industries and livelihoods have also been forced to cope with reduced access to water. The drought has also raised important questions about sustainability long-term. Partial replenishment during the 2016 rainy season did not eliminate the crisis, leaving California’s water supply in the red [18]. In the future, economic and demographic growth seem set to vaunt demand for water far beyond available supply.

### Socio-communicative responses to drought in an agenda-setting context

Droughts and other chronic environmental crises generate a complex timeline for public response as their effects, even when anticipated, may not be recognized as indicative of a crisis for some time. Droughts themselves emerge as chronic only after multiple cycles of water store depletion followed by lack of replenishing precipitation [21]. For this reason, the public generally hears about rather than directly experiences drought. Media and social media flows move at different speeds: Available evidence and amount of public concern possess different specific masses and vary in “viscosity,” that is, in their ability to rapidly spill into new areas of public concern [7]. Information channels can be quite circuitous and the coupling between environmental and social phenomena tenuous if not tangibly problematic. Even when public conversations develop, the complexity involved in drought response tends to leave communications fragmented and unsatisfactory for a public eager to return to normal activity. Because policymakers frequently respond to water scarcity by instituting restrictions, public response may reflect actions of policymakers rather than the drought itself [22, 23]. Agenda-setting communication pathways reveal that the relationship between scarcity and media coverage involves a recursive, non-linear web of causes and effects [24]. Yet, the existing body of literature has yet to identify the communicative function(s) of generalized, social media-based conversations about a chronic crisis in contrast with the communicative function(s) of traditional media coverage. There is uncertainty concerning the order in which public response approaches critical mass in different communication channels. For example, the most recent and notable Su and Borah study, focuses on a limited period incident (a public policy announcement), following it within a window of a few days. By contrast, our study looks at a chronic crisis, stretching over several months and observing the data as a continuous flow, which better allows determining order of precedence.

#### Agenda-setting

Agenda-setting theory emerged during the 1970s and originally stated that issues made salient by the news media would soon reach the same level of salience in the minds of news consumers [3, 25]. Subsequent research specified the contingent conditions that enabled the transference of issue salience, identifying need for orientation, relevance, and uncertainty as foundational to agenda-setting [26–28]. Today, as during the 1970s, audience interest in an issue and uncertainty regarding the issue combine to instigate increased information-seeking. The more personally relevant consumers perceive an issue to be, the more they will desire information to reduce their uncertainty regarding theissue [29] Chapter 2. The more the media serve as sources of information, the more audiences will attend to the issues, issue attributes, and issue evaluations privileged by the sources in question [26].

Yet, the information environment of today bears little resemblance to that of the 1970s. The dramatic increase in availability of sources of information [1] has turned the relationship between source and receiver into a two-way street, where the influencer and the influenced may change places [30]. Internet access gives media consumers the agency to select news sources from an ever-expanding array of online platforms, many of which provide social connectivity in addition to information [31]. Furthermore, media consumers actively re-disseminate the news, at times becoming significant media players [32]. In this context we need to consider that every individual with an Internet connection can frame public issues, disseminate public affairs content, and source public discourse within the digital space, powers formerly available only to media organizations. As agenda-setting processes rely upon the human quest for perceived relevance and need for orientation, the ubiquity and sociability that characterize online news experiences may be particularly suited to this quest [32].

More recent conceptual elaboration of agenda-setting processes [3] supports the notion of a digital information environment wherein the agenda of the ordinary consumer (citizen) may be proactive and not simply reactive. McCombs defined reverse agenda-setting as a journalistic response to public concerns, real or perceived. As such, the reverse agenda-setting process gives rise to the question of which contexts and contingent conditions are most likely to induce citizen push-back by what some researchers call “digitally connected publics” [7, p. 195]. Attempting to determine the strength of the two-way relationship between the public and media prompts further investigation, especially by time-order analysis of content publication, into how and when content authored by these members of public wields influence over the “broader public and media agendas and the framing of public issues [7, p. 195]. Patterns of “leading and lagging indicators,” which can be explored by statistical analysis of social and traditional media production, provide quantifiable evidence for symbiotic agenda-setting in the digital era [7, p. 209]. Stated another way, we need to be mindful of the fact that agenda setting can now be captured by the observation of news flows at the macro level. Our study builds and extends on the immediately preceding literature mentioned above that examined similar phenomena [6, 8, 11] is at least two ways. First, we use a longer temporal perspective. Second, we observe the interaction between social and traditional media as continuous process, which detects any possible recurring processes. Finally, we consider the role of broader information seeking (search engines) in the agenda setting process.

Combined, our three approaches can determine in a more accurate way if activity spikes in social media (e.g., tweets) co-occur with other phenomena (water depletion, media coverage, Google searches) we can determine if social media precedes or follows other communicative or environmental phenomena. This aligns our research with the quest for broader patterns identified as gaps in the preceding literature [7]. At the same time, our approach connects to some recent research that has taken pains to illuminate the topical similarities between what Internet users are talking about online. and which issues are being covered and in what temporal order by their local mass-media outlets [6, 8, 11]. The overall theoretical conclusions of existing research are not, however, definitive. Neuman et al. [7] point to the fact that the jury is still out regarding whether social or traditional media are at the vanguard of the agenda-setting process, while Su and Borah [8] only detected short term, reversible effects in the impact of social media on traditional media. This constitutes a specific theoretical gap in the literature that our research attempts to address.

#### Agenda-setting in chronic crisis situations

We have taken pains to justify the need for a macro lens when studying agenda-setting processes in social and traditional media, referring to the relevant literature [6, 8, 33]. The focus now is on agenda-setting in crisis situations specifically, with especial emphasis on discovering an order of precedence between formal media and informal social media or search behaviors. While some work has been conducted in this area [34, 35], most of it remains at the highest level of abstraction, asking broad questions. In view of this, our work articulates theory-based more specific approaches, which represents a novel contribution. Given the high levels of uncertainty and perceived relevance that attend crisis situations [36]we propose that social media and information seeking via online tools may prove especially salient in times of crisis. For those in affected areas, concern for self and surroundings may manifest as extreme anxiety [37]. This may lead to seeking and acquiring information by whatever means available, even before media crystalizes a definitive picture of the situation [38]. Immediacy of information acquisition is also justified by the state of ambiguity in which consumers and citizens find themselves. It is also important to note that ambiguity enhances crisis-related anxiety. Previous research also suggests [39, 40] that duration of a crisis directly affects public perceptions of the current threat level [41]. It follows that persistent crises, such as droughts, might produce some the highest levels of anxiety and, therefore, may trigger early and massive information-seeking behaviors among potential victims [1, 2].

Information seekers in today’s hyper-connected information environment—already reeling from “abundance and cognitive surplus”—naturally turn toward the obvious and most convenient sources of information: search engines [33, 42]. Combined with the anxiety and ambiguity of crisis, needs for orientation expand and intensify, rendering them easier to satisfy [39, p. 4].

At the same time, individuals whose anxiety has been triggered by a crisis situation will add to their information journey as well as employ other data collection strategies [2]. Starting with information gleaned from search engines, individuals continue collecting extra information, often re-sharing and producing new interpretations of that same information through social media. While social media constitutes only one aspect of online activity, its function as a real-time, open-access, geographically specific information nexus may help “develop a shared culture in crisis” [43], further intensifying perceived relevance and social need for orientation among users [26, 44]. Overall, these processes can lead to more immediate and individualized information-seeking and dissemination behaviors, which trigger early communicative responses to the crisis. These responses tend to follow a path of least resistance [1]. Information will be collected and disseminated through the means that are most convenient and easy to use. In many situations, these consist of social media channels, wherein individuals are already immersed in the platform as a matter or social custom and cultural preference. In other situations, mere web browsing with some assistance from Google, or another search engine may be a means of first recourse and it capable of acting as an agenda-setting factor.

## Method

Research on the mutual dependency of traditional and social media communication channels and intersections with search-engine-based information-seeking should consider the fluctuations in orders of priority of salient issues in accordance with the degree of ambiguity or anxiety in each communicative situation. While the theoretical agenda-setting perspective has been well developed, relatively little empirical evidence exists regarding the ways in which search engine use or social / traditional media interact, especially when we deal with a chronic crisis.

The California water crisis, which has been thoroughly documented in social and traditional media as well as the records kept by Google on search patterns, serves as a valuable vantage point from which to address these provocative questions. In a way, the crisis may be considered as a natural quasi-experiment, where the objective crisis (water depletion) is the stimulus that activates a variety of communication responses occurring in a certain order of precedence. The time-order of Twitter activity and search-engine-based information-seeking in response to the crisis may be viewed as artifacts of a societal agenda-setting process. Two research question and a hypothesis were formulated along these lines.

RQ1: Which communication elements (social media conversations, search behaviors, and traditional media coverage), and in what order, respond to the California drought crisis?
RQ2: What drives the intra-media agenda as suggested by order of response?
H1: Social media precede traditional media coverage of the crisis.

**Fig 1:**
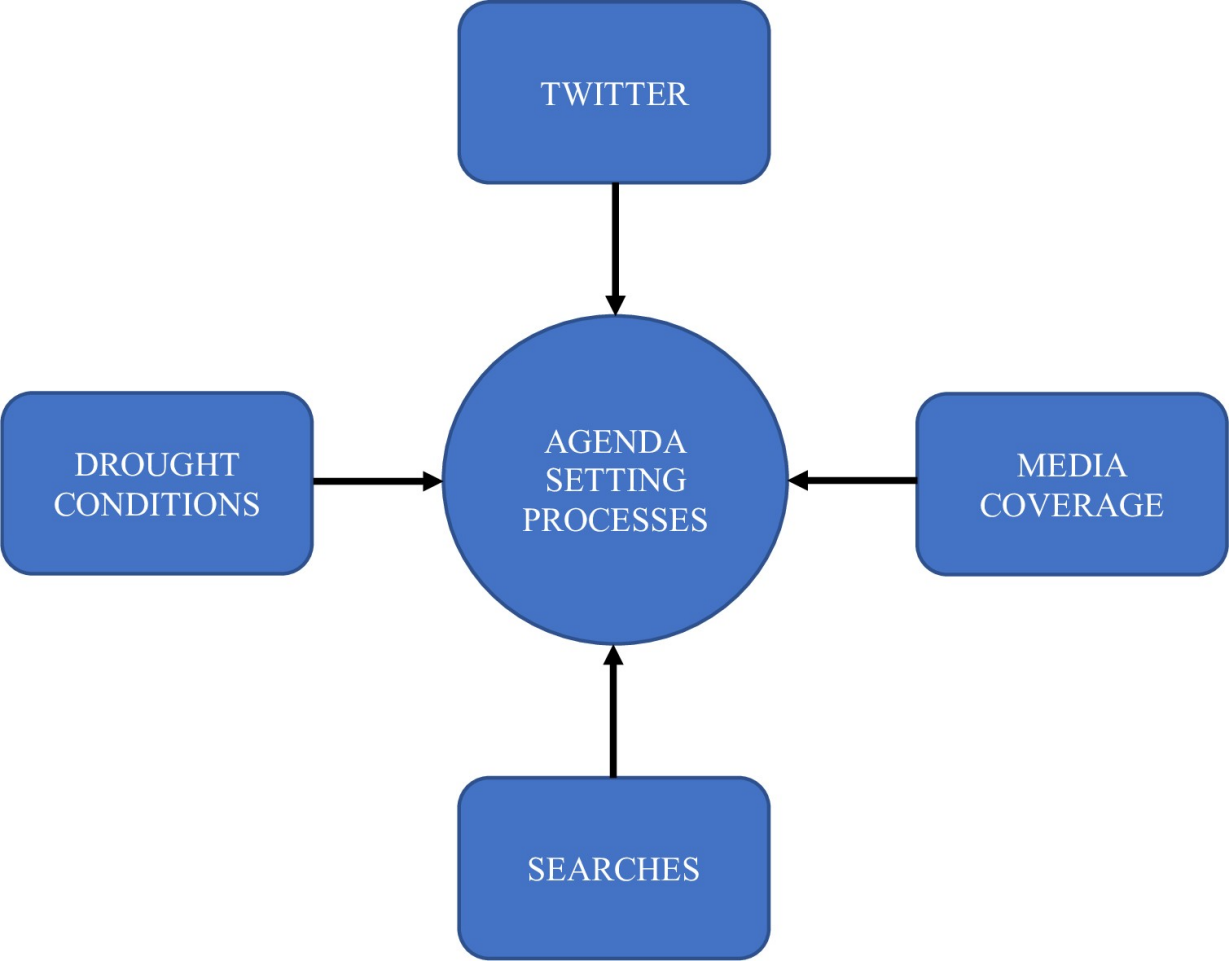
Interactions between communication elements in the California drought are currently undefined.

**Fig 2:**
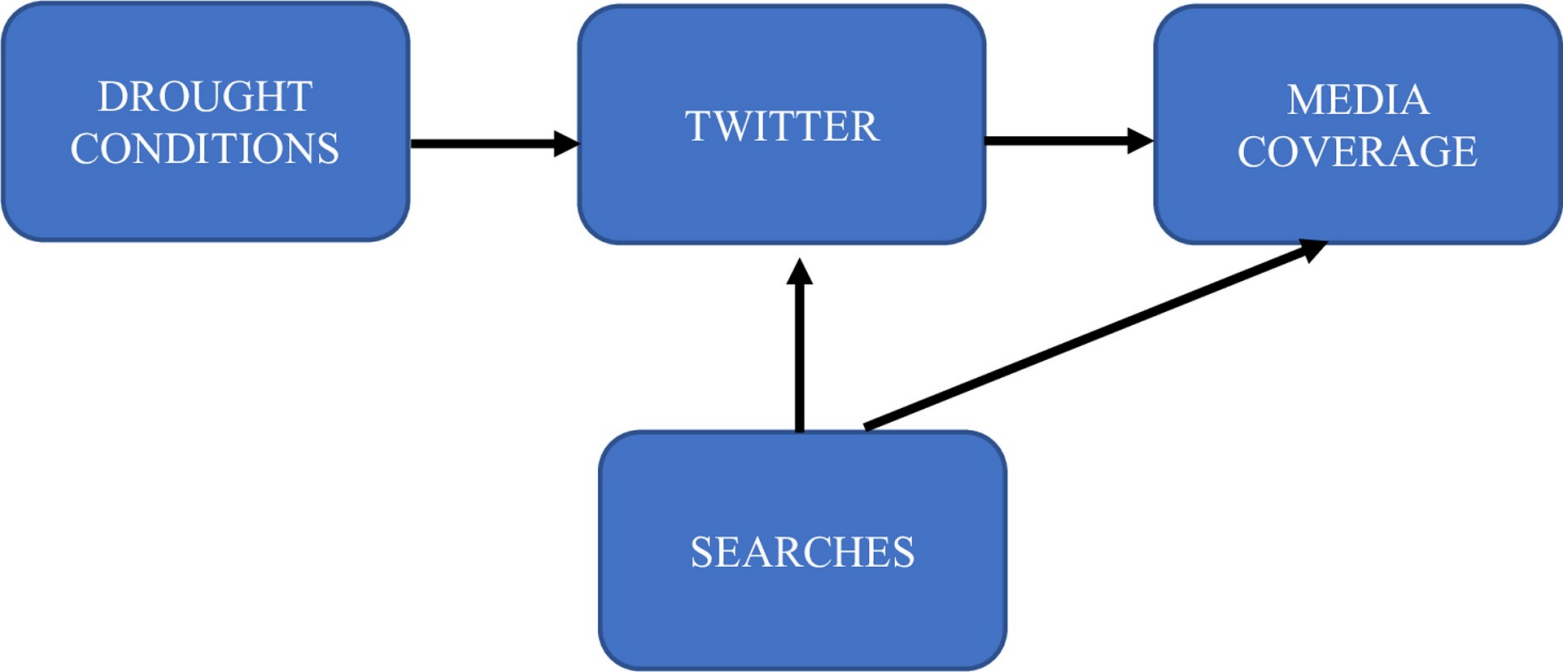
Theory-based presumptive interactions between communicative factors during the California drought.

Our model aims to chart the relevant communication pathways to pierce the cloud of uncertainty surrounding “agenda-setting processes” (Fig 1). The research questions were formulated with the expectation that social media will *answer first* and will be *reinforced by Google* searches (Fig 2).

### Data collection

To answer research questions and establish order of precedence, we collected data about the severity of the drought as observed in mediated spaces from February 2013 through September 2015. Drought severity in the San Joaquin River Valley, the area of California most impacted by the drought and whose impact drove the public conversation, was measured using the Standardized Soil Moisture Index, or SSI [45]. SSI in a variant of the precipitation index, which uses streamflow instead of direct precipitation measurements. Streamflow is captured form rivers, streams, and other flowing bodies of water, which more accurately captures the precipitation that was directly captured by the soil. Regardless of the measurement method, the two indices are nearly identical, capturing the same data, which is the amount of water that is available in an area. The standardized soil moisture index is preferable because it is weighted toward capturing land drought severity.

In measurable terms, three-month SSI was calculated at five randomly selected locations using the soil moisture data obtained from NLDAS, NASA’s North American Land Data Assimilation Systems dataset [46]. Hourly soil moisture data from NLDAS spanning January 1980 through September 2015 was converted to monthly time series and entered the Standardized Precipitation Index calculator [47]. Output containing the three-month SSI values was then used for further analysis. It is important to note that while SSI measures the severity of the drought, it provides only a baseline measure of how dramatic the real-life conditions were at each point during the crisis. However, by introducing the measure in the predictive model we do not assume that the media or information consumers reacted directly and consciously to it, since the information was not publicly known. Rather, we consider that the worsening objective draught conditions created the environmental circumstances felt through a variety of personal experiences, especially dwindling water supplies in the homes and fields, that led to media concerns. SSI offers an assessment of the magnitude of the objective water shortage was, which created the backdrop of the crisis. SSI is the ultimate ground truth, against which we measure everything else, not a subjectively known quantity to which the human actors responded consciously.

We assessed the mediated response to the drought via volume of Google searches related to the drought and Twitter activity and media coverage of the California water crisis. We collected data using a query algorithm with core strings “California water” and “California drought.” From Google Trends, we retrieved the volume of searches in California for “California Water” and “California Drought” for searches performed in California, which was the highest level of granularity available for this data. (Google can release a standardized index for search volume for any given period. The index is standardized on a 0–100 scale around the highest volume detected during the period of interest [48]. Thus, the week with the highest volume is scored at 100, while everything else is calculated as a fraction of that week’s score. (This method of standardization is intended to protect Google’s commercial interest in the raw data.)

Using the Twitter Application Programming Interface (API), we retrieved the query ((climate OR drought OR water) (california OR ca)) OR cawater OR cadrought OR saveourwater. The query was more complex because it included several hashtags specific to Twitter (cawater, cadrought, and saveourwater). The pilot sampling process revealed that “climate” was also associated with the California drought crisis, further adding to query complexity. The query was limited in space to central California tweets geo-located by meta-data or in-text geographic identification. A total of 186,149 tweets were retrieved. The traditional media outlet selected was newspaper *The Modesto Bee*, an important regional newspaper covering the San Joaquin River Valley, the area most impacted by the crisis. The online newspaper archive was searched for articles containing the strings “California water” and “California drought” and 1,853 articles were retrieved.

Newspaper and Twitter data was cleaned for relevance and location. Non-relevant items were identified through hand-coding of a random sample of the collected items. We then applied a Multinomial Naive Bayesian algorithm to extend the classification made in the hand-coded items to the entire dataset. A Multinomial Naive Bayesian classifier uses word frequencies to characterize each document and then classifies the documents by comparing the similarity between frequency of word occurrence across documents (in this case articles of tweets). The averages of each accuracy metric with each class weighted to their prevalence in the data were:

Precision, .77, recall, .72, and F1, score .68, which are within the typical limits for reliability of this type of analysis. This is a widely used method, which provides robust results [49] avoiding skewing the dataset with tweets related to water-heater troubles or water crises in other regions than California.

### Data analysis

**Fig 3:**
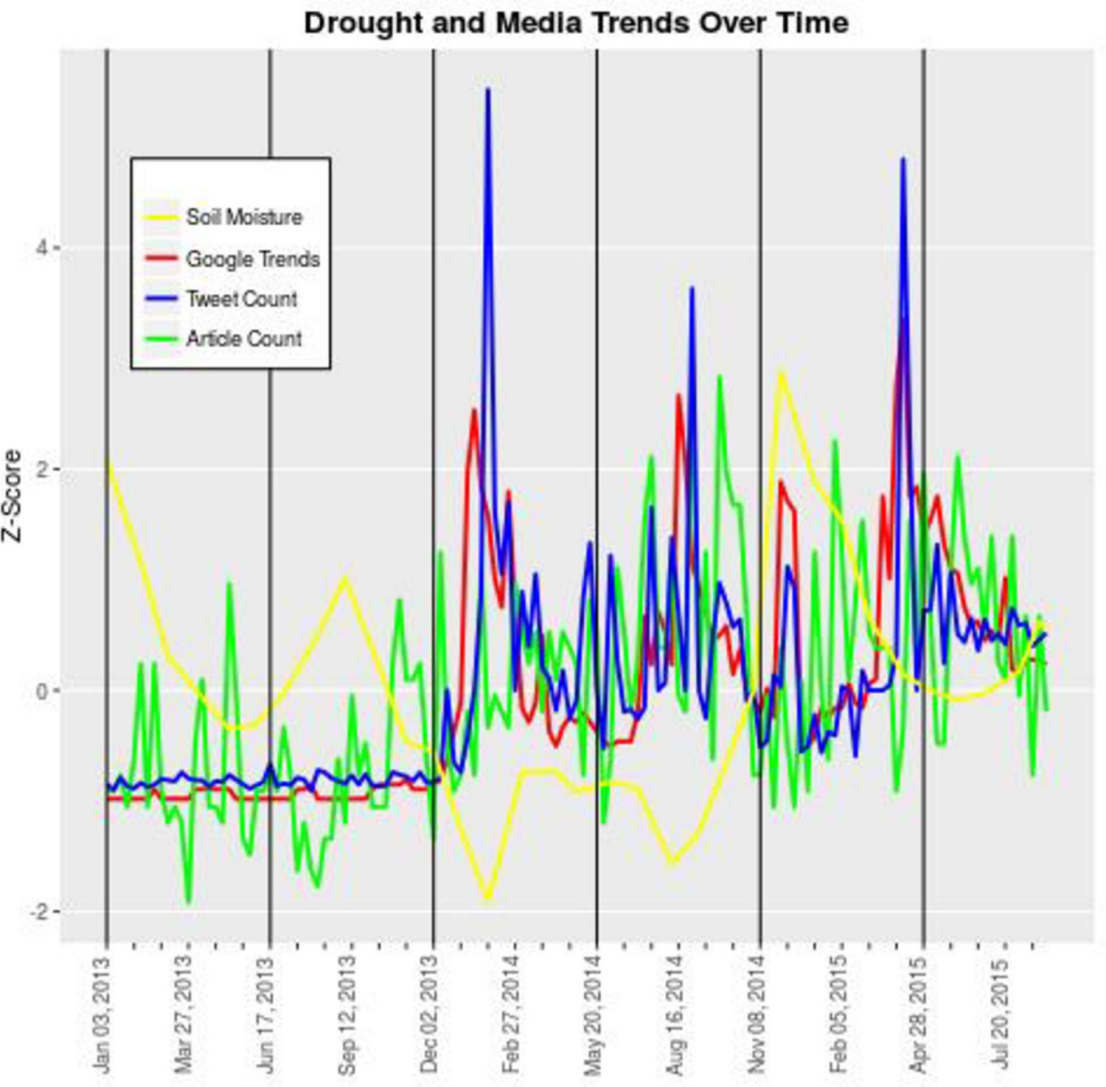
The Standardized Soil Moisture Index, or SSI series representative of the geological conditions, was first analyzed in a pairwise manner with each of the media data series. The aim was to determine a baseline understanding of the pairwise relationships between all variables (soil moisture, Twitter activity, search engine trends and media coverage). Results illustrated which of the three media categories, and to what degree, responded to hydrological conditions.

To ascertain the relationship among these four trends, a mix of time-series analysis approaches were used. The Standardized Soil Moisture Index, or SSI series representative of the geological conditions, was first analyzed in a pairwise manner with each of the media data series (see Fig 3).

The aim was to determine a baseline understanding of the pairwise relationships between all variables (soil moisture, Twitter activity, search engine trends and media coverage). Results illustrated which of the three media categories, and to what degree, responded to hydrological conditions. Once the baseline relationships were established, we analyzed the order of precedence in media responses to changes in hydrological conditions.

Due to the non-stationary nature of the SSI series, we first built an auto-regressive-moving-average (ARMA) model of the SSI series and then applied the resulting filter to both the SSI series and the media series under consideration. The cross-correlation function of the filtered data series was then examined for potential relationships. Large spikes in the cross-correlation function (CCF) indicated lags in the SSI series that might potentially influence the media series. Therefore, linear regressions on the media series were run using the identified lags. However, examination of the residuals of these regressions indicated auto-correlation. Thus, an ARMA model capable of accommodating this error-term structure was constructed for each data series. We were then able to determine if there was a statistically significant relationship between geological conditions and activity in Google searches, media coverage, and number of tweets. In the second phase of analysis, the sequential relationship (order or precedence) between the different media was determined by means of vector auto-regression (VAR) analysis. Because the media data series are stationary, a simple VAR could determine which media series, if any, influenced the outcomes of the other data series. Following a simple diagnostic test to determine which order of VAR was appropriate, an examination of the VAR results for statistically significant coefficients yielded evidence for precedence among the data series.

## Results and discussion

**Fig 4:**
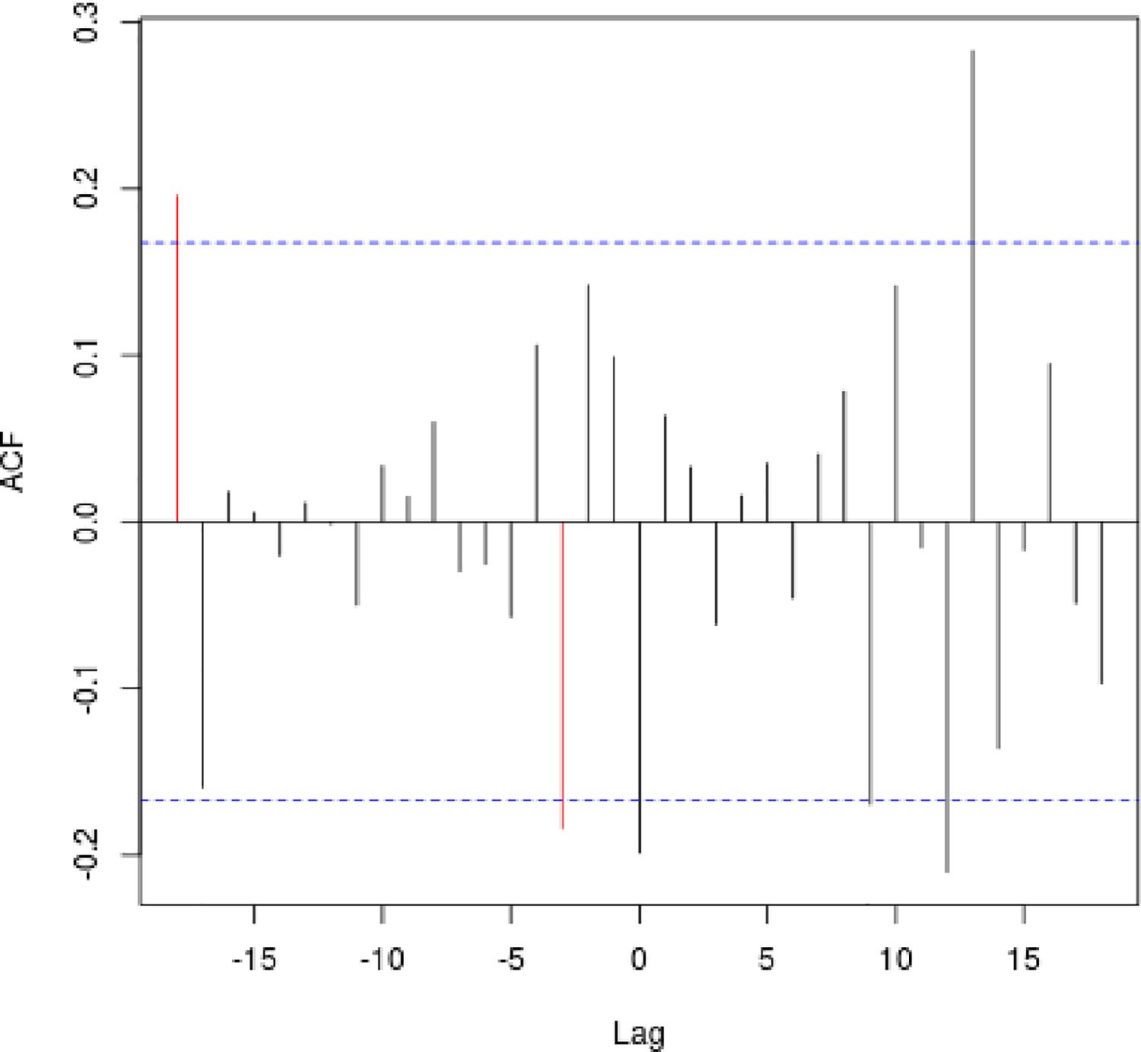
Correlation between lagged drought severity and twitter activity.

An analysis of association and temporal precedence between geological data (soil moisture index) and the media data series revealed that only Twitter data was significantly associated with declines in water supply (see Fig 4). Cross-correlation function analysis (CCF) for media coverage and Google search trends indicated no significant results; the lagged regression procedure was not warranted.

The CCF analysis for the relationship between soil moisture and tweeting trends showed several peaks. While there were several potentially relevant lags in the SSI data, only the three-week lag had a significant relationship with the Twitter data series, indicating that Twitter activity reacted to changes in hydrological conditions after a delay of just under a month. The negative sign of the coefficient for lagged SSI data series in Table 1 indicates that, as the severity of the drought increases, the number of drought-related tweets increases as well—we know that the Standardized Soil Moisture Index declines as the drought worsens. The magnitude of the coefficient indicated that an increase of one standard deviation away from the mean in drought severity resulted in a .343 standard deviation increase in Twitter activity. The remaining coefficients in Table 1 were necessitated by the structure of the Twitter data series, which is most closely approximated by an ARMA (1,2) process; they were included for control purposes. The residuals of this regression follow a white-noise process. As such, the standard-error estimates of the model are valid.

**Table 1:** Drought severity and Twitter activity.

	Dependent variable:
	Twitter Activity
Twitter 1 Week Delay	0.979***
	-0.026
Error Term 1 Week Delay	0.620***
	-0.095
Error Term 2 Week Delay	—0.230**
	-0.097
Constant	0.032
	-0.381
Standardized Soil moisture Index 3 Week Delay	-0.343***
	-0.119
Observations	139
Log Likelihood	-163.945
Sigma square	0.616
Akaike Inf. Crit.	339.889

Note: *p<0.1

**p<0.05

***p<0.01.

In step two, as mentioned, we performed a VAR of the three media data series to determine the order of precedence between the media variables themselves. However, as only Twitter activity was found to respond causally to the soil moisture indicator (SSI), the order of precedence is media-relative. In other words, as Twitter was the only medium that responded to soil moisture depletion, VAR analysis revealed how tweeting co-evolved with other media. Specifically, we asked whether tweeting itself catalyzed the public drought conversation and whether Twitter interacted with the other two communication elements in question.

VAR analysis generated linear models for each data series indicating the relationship between that series and the one-week lag of all four data series. As shown in Tables 2–4, the key finding is that Google searches influenced both Twitter activity and the number of newspaper articles. Diagnostic tests (Akaike Information Criterion, Bayesian Information Criterion, etc.) indicated that a VAR(1) model was most appropriate.

**Table 2:** Temporal effect of Google search behavior on Twitter and newspaper coverage.

	Dependent variable:
	Google Trends
Google Trends 1 Week Delay	0.846***
	-0.06
Article Count 1 Week Delay	0.071
	-0.049
Twitter Activity 1 Week Delay	-0.022
	-0.062
Constant	0.008
	-0.044
Observations	138
R-square	0.742
Adjusted R-square	0.736
Residual Std. Error	0.516 (df 134)
F Statistic	128.276*** (df = 3; 134)

Note: *p<0.1; **p<0.05

***p<0.01.

**Table 3:** Temporal effect of Twitter activity on Google search behavior and newspaper coverage.

	Dependent variable:
	Twitter Activity
Google Trends 1 Week Delay	0.664***
	-0.081
Article Count 1 Week Delay	0.059
	-0.065
Twitter Activity 1 Week Delay	0.057
	-0.084
Constant	0.007
	-0.059
Observations	138
R-square	0.534
Adjusted R-square	0.523
Residual Std. Error	0.694 (df = 134)
F Statistic	51.126*** (df = 3; 134)

Note: *p<0.1; **p<0.05

***p<0.01.

**Table 4:** Newspaper coverage effect on Google search behavior and Twitter activity.

	Dependent variable:
	Article Count
Google Trends 1 Week Delay	0.318***
	-0.101
Article Count 1 Week Delay	0.301***
	-0.081
Twitter Activity 1 Week Delay	0.023
	-0.104
Constant	0.007
	-0.073
Observations	138
R2	0.279
Adjusted R-square	0.263
Residual Std. Error	0.862 (df = 134)
F Statistic	17.258*** (df = 3; 134)

Note: *p<0.1; **p<0.05

***p<0.01.

The importance of the Google searches in influencing the other elements is evidenced by the statistically significant effect of the search trends on each of the other data series. In addition, the first lag of the Google search data series had the largest coefficient in each of the regressions. The Google series itself was highly autoregressive, with the previous week’s value highly correlated (.846) with the current week’s value. In other words, Google search behavior is time resilient. Search patterns do not develop in a week and then go away. Neither do Google searches come and go randomly. Rather, they build on past user interest and grow in intensity over time.

Turning to the remaining series, a one standard deviation increase in Google searches from the previous week results in almost two-thirds of a standard deviation increase in the number of Tweets. For newspaper article count, there was an almost equally strong relationship between the number of articles published on the drought, the previous week’s article count and the previous week’s search activity. For each of these series, a one standard deviation increase resulted in an almost one-third standard deviation increase in the number of published articles about the drought.

In terms of agenda-setting theory, results from step one and step two indicated that 1) the public conversation about water depletion in the soil was driven by Twitter alone, 2) both tweeting and media coverage are driven by Google searches, which is the communication element with the strongest intra-media agenda-setting effect 3) social media and traditional media do not directly influence each other. We turn now to our guiding research questions.

RQ1: Which and in what order of precedence do communication factors (social media, search behavior, and traditional media) respond to a crisis?

Only Twitter significantly and proximally (three-week lag) responded to physical indicators of the drought. This indicates higher Twitter sensitivity to drought conditions. Twitter responded to the drought at a finer level of granularity.

RQ2: What drives the intra-media agenda by order of response?

In media space, Google searches drive both tweeting and traditional media activity. Google is the core, inter-media agenda-setting factor. The fact that Google searches are not directly triggered by the drought conditions only emphasizes the inter-media effect of information-seeking behavior. Google search seems to be a constant, “low-frequency” signal that dominates the information landscape, shifting according to other likely social and psychological motivations.

H1: Social media precede traditional media coverage of the crisis.

**Fig 5:**
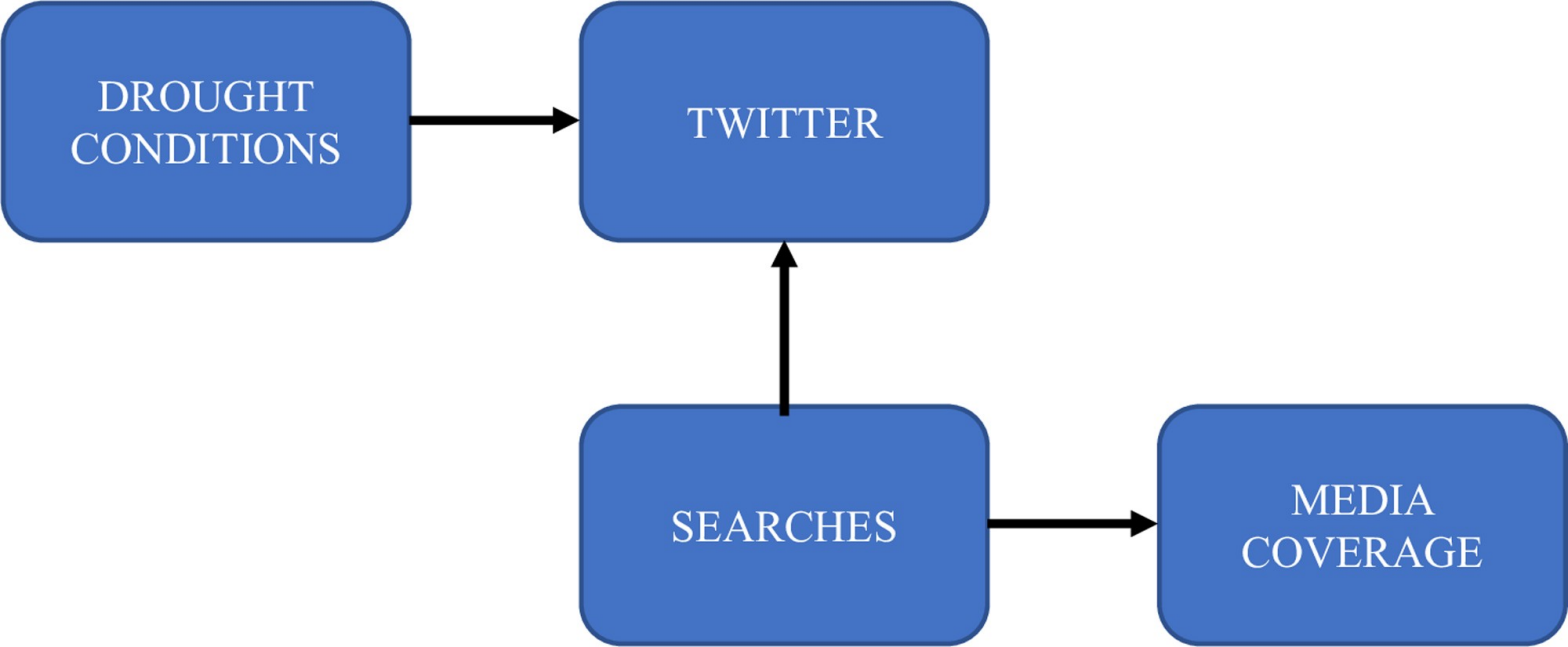
The drought crisis agenda appears to be set by Twitter. Google searches reinforce Twitter activity as well as media coverage, although neither of them are directly responsive to drought conditions.

The final model of interaction and agenda-setting processes is shown in Fig 5. Social media (Twitter) does not lead or lag traditional media. They both follow their own paths. Thus, hypothesis 1 is not confirmed, However, this negative result is highly significant in its own way, in that it suggests that Twitter is attuned to changes in the natural conditions, while traditional media is not. Furthermore, both social and traditional media are influenced by Google searches, a factor underexplored in agenda-setting work and research addressing the connection between communicative and physical systems (see Fig 5).

## Summary and conclusions

The present study reveals that social media, particularly Twitter, reacted relatively promptly (within three weeks) to a chronic environmental crisis, namely the California drought of 2013–2015. Framed as an inter-media agenda setting problem, our study assessed the centrality of information-seeking via search engines in the contemporary media environment. While Twitter was an “early responder,” trends in tweeting were predictable and reinforced by Google searches. Media coverage at once trailed and was predicted by Google searches. The chain of effects identified by the study may be summarized as follows: While Twitter users were the first, and, in fact, only elements in the study that reacted promptly to a worsening natural crisis, Twitter activity breathed in and out with the Google search activities of people exposed to the crisis. Media coverage also ebbed and flowed in rhythm with Google searches. Yet we did not find search behavior to be directly connected to the crisis. Instead, it was more closely tied to social media behavior. As mentioned above, we need to qualify these finding and observations by stating that media and information seeking responses were not due to public knowledge of SSI declines, but due to the felt water shortage. The SSI measures how the severity of the water shortage, which was in turn experienced by California residents and farmers as dwindling water supplies in the homes and in the fields.

Our findings suggest that, at least in chronic crisis situations, social media, such as Twitter, play an important role. In response to environmental stressors, Twitter was found to be a sensitive and reactant medium. The public response was relatively immediate (three weeks) given the chronic nature of the problem. We further found that search engines worked in tandem with social media, feeding social-media activity. We note, however, that search-engine behavior only had a relationship to social media behavior. Public response to the chronic stressor was social-media dependent; search engine behavior independently impacted the social media response. Newspaper coverage was least sensitive to drought conditions but was related to Google searches. This suggests that, in this environmental crisis, social media was the agenda-setting communication channel. Practitioners and scholars alike would do well to explore the implications of this finding more in depth. For example, journalists should monitor in a professional manner, using advanced analytics tool, such as social media listening media for trending conversations and current searches associated with environmental stressors. Tools such as Google Trends and Google Alerts, as well as advanced tools, such as Synthesio (https://www.synthesio.com/blog/measuring-growing-impact-climate-change-social-media-monitoring-tools/) can offer unique insights into what is happening in the world outside the newsroom. More important, journalists should spread the coverage of chronic crises over time, coming back to it periodically and topically, such that they are not caught unawares by sudden changes in the public opinion. Practicing healthy community-based journalism and engaging the community in townhall conversations or other creative social media campaigns, such as “citizen briefings,” modeled after the Obama administration Citizen Briefing Book (https://obamawhitehouse.archives.gov/sites/default/files/microsites/Citizens_Briefing_Book_Final2.pdf), where the public is asked to shape future conversations, could be another approach.

Previous studies have examined ideas such as potential correlation between Google trends for searches and media coverage of various issues. Rojecki and Meraz [13] found strong correlation between Google searchers and media coverage of dubious claims about political actors during the 2004 presidential electoral cycle. Similarly, Google searches were found as reflections and salience measures of media coverage [14, 16]. Our work adds to this line of research several new insights. Rojecki and Meraz (2014) focused mostly on misinformation, rather than on factual, valid news go in the same direction as ours, the present study is more sensitive to the coverage of real, factual news. Langer and Gruber [16] used Google Trends as an indicator of public attention, while Mellon [14] turned to this indicator as a proxy for media salience. Our work is methodologically more complete, as it connects four different factors, each of them seen as a potential force shaping public opinion: natural stressor, Google search behaviors, media consumption, and social media interactions. In fact, our work is closer related to flu and epidemiological data of flu outbreaks [50], which correlated flu (a natural stressor) with Google searches and determined a close matching of the two time series. At the same time, our paper pinpoints Google searches as fuel for online activity even without direct relationships to environmental stressors, which is unexpected and yet understandable. Our findings Google is, after all, the go-to source of information for internet users today. It dominates the search engine market, facilitating 63.8 percent of desktop searches [51]. Of course, introducing Google searches into the media equation should only be done with great caution. It was not Google content or service that led the agenda-setting process here. It was user behavior. Of the behaviors that set the agenda, it was not using social media or reading the newspapers or watching the news that led to the public conversation. It was search-engine-based information seeking. At the same time, the influential channel reacting most quickly to the crisis was Twitter.

Although offering a new perspective on the interaction between social media, search, and old media, our study is only a beginning in this direction. Our study is limited in a few ways. Methodologically, to assess which types of content lead the conversation, a targeted set of research questions coupled with the appropriate methodologies must be deployed. Future research might consider looking in detail at the nature of the content accessed by individuals and the sources from whence it came. Seen from this perspective, inquiries might expand to incorporate all types of communication elements, including user-generated reference materials, such as Wikipedia, Buzzfeed and Reddit. Similarly, analysis of primary as well as hybrid information sources, such as press agencies, media organizations and social media actors, might extend the applicability of our finding still further. The available resources prevented us from conducting a large, national study of interactions between these factors. As a national, global even, issue, water shortage and its impact on the economy and society can and should be studied over longer periods of time and with a broader scope. We hope that our study serves as a helpful building block for other scholars grappling with the contemporary media-verse in its many environmental, social, and cultural contexts.
